# Evaluation of 511 keV photon attenuation by a novel 32-channel phased array prospectively designed for cardiovascular hybrid PET/MRI imaging

**DOI:** 10.1186/s41824-020-00076-w

**Published:** 2020-05-12

**Authors:** Adam Farag, R. Terry Thompson, Jonathan D. Thiessen, Heather Biernaski, Frank S. Prato, Jean Théberge

**Affiliations:** 1 Imaging Division, Lawson Health Research Institute, London, Ontario Canada; 2 Department of Medical Biophysics, Western University, London, Ontario Canada; 3 Department of Medical Imaging, Western University, London, Ontario Canada; 4 Diagnostic Imaging, St. Joseph’s Health Care, London, Ontario Canada

**Keywords:** PET/MRI, Cardiac imaging, Phased array, Attenuation map, Attenuation correction

## Abstract

**Background:**

Simultaneous cardiovascular imaging with positron emission tomography (PET) and magnetic resonance imaging (MRI) requires tools such as radio frequency (RF) phased arrays to achieve high temporal and spatial resolution in the MRI, as well as accurate quantification of PET. Today, high-density phased arrays (> 16 channels) used for cardiovascular PET/MRI are not designed to achieve low PET attenuation, and correcting the PET attenuation they cause requires off-line reconstruction, extra time and resources.

**Purpose:**

Motivated by previous work assessing the MRI performance of a novel prospectively designed 32-channel phased array, this study assessed the PET image quality with this array in place. Guided by NEMA standards, PET performance was measured using global PET counts, regional background variation (BV), contrast recovery (CR) and contrast-to-noise ratio (CNR) for both the novel array and standard arrays (mMR 12-channel and MRI 32-channel). Nonattenuation-corrected (NAC) data from all arrays (and each part of the array) were processed and compared to no-array, and relative percentage difference (RPD) of the global means was estimated and reported for each part of the arrays. Attenuation correction (AC) of PET images (water in the phantom) using two approaches, MR-based AC map (MRAC) and dual-energy CT-based map (DCTAC), was performed, and RPD compared for each part of the arrays. Percent mean attenuation within regions of interests of the phantom images from each array were compared using a two-way analysis of variance (ANOVA).

**Results:**

The NAC data of the anterior part of the novel array recorded the least PET attenuation (≤ 2%); while the full novel array (anterior and posterior together) AC data, produced by MRAC and DCTAC approaches, recorded attenuation of 1.5 ± 2.9% and 0.0 ± 2.5%, respectively. The novel array PET count loss was significantly lower (*p* = 0.001) than those caused by the standard arrays.

**Conclusions:**

Results of this novel 32-channel cardiac array PET performance evaluation, together with its previously reported MRI performance assessment, suggest the novel array to be a strong alternative to the standard arrays currently used for cardiovascular hybrid PET/MRI imaging. It enables accurate PET quantification and high-temporal and spatial resolution for MR imaging.

## Introduction

Simultaneous positron emission tomography (PET) and magnetic resonance imaging (MRI) are a valuable tool for assessing different forms of cardiac diseases using PET tracers sensitive to perfusion (13 N-ammonia), inflammation or myocardial viability (18F-FDG), or sympathetic innervation (11C-HED) in combination with MRI measurements of fibrosis, function and more. However, this value is tied to how accurately PET measurements can be quantified and interpreted. A recent joint position statement from the European Society of Cardiovascular Radiology (ESCR) and the European Association of Nuclear Medicine (EANM) (Nensa et al., 2018) pointed out some unsolved issues of PET quantification in cardiovascular hybrid PET/MRI imaging. The statement identified the need for high-temporal and spatial resolutions for imaging of coronary atherosclerosis and other heart diseases. While accurate PET quantification is an ongoing topic of research, the root cause of inaccurate quantification is usually associated with inaccurate attenuation correction (AC) inflicted by one or more of the following: the method/models used for PET attenuation correction (Catana et al., 2010; Ghadiri et al., 2011; Marshall et al., 2013; Wagenknecht et al., 2013), natural motion due to patient respiration and heart activity (Huang et al., 2014; Kolbitsch et al., 2014; Lindemann et al., 2019) and registration of flexible hardware (Eldib et al., 2014; Eldib et al., 2015; Fürst et al., 2012; Kartmann et al., 2013; MacDonald et al., 2011; Paulus et al., 2012). Notably, one of the causes of inaccurate quantification of PET is the radio frequency arrays/coils (mobile, i.e. endorectal prostate, and/or flexible, i.e. body-matrix anterior), which are required to achieve high-temporal and spatial resolution in cardiovascular imaging. Photon attenuation from rigid and fixed arrays can be accurately corrected for, using a dual-energy computed tomography-based AC map (DCTAC) (Patrick et al., 2017); however, one must consider the accuracy of the derived linear attenuation coefficient (LAC) at 511 keV.

The effect of a fixed phased array on PET quantification has been studied extensively (MacDonald et al., 2011), and it is reported that PET tracer activities were underestimated by 19% when the AC map was not applied. Even when flexible phased arrays are designed to minimize attenuation, deviations in PET quantification as large as 10–15% were reported in regions of interest adjacent to the flexible phased arrays (Kartmann et al., 2013; Paulus et al., 2012). Thus, applying AC map for flexible arrays is ideal for accurate PET quantification in most applications. Thereafter, more studies were dedicated to establishing a method to correct for attenuation of flexible arrays and in particular the registration of the AC map to PET images. A method for accurate registration of AC map of flexible RF arrays was developed and reported using fiducial markers to guide the registration of the hardware AC map with the PET image (Kartmann et al., 2013; Paulus et al., 2012). Most recently, the AC maps of flexible arrays were spatially registered using alternative methods without the need for markers: (1) an automatic algorithm using data from an ultra-short echo time (UTE) MRI scan (Eldib et al., 2015) and (2) a method using the Microsoft Kinect V2 depth camera to track the 3D surface of the RF array and localize it inside the scanner’s FOV and determine hardware AC map deformation parameters (Frohwein et al., 2018). However, such approaches could not address the full range of imaging conditions, for example, the marker methods require markers to be present and fixed on the array during PET acquisition, which could affect the MRI images, besides may cause inaccurate registration which may result to inaccurate quantification of PET (Eldib et al., 2016). Meanwhile, offline algorithms used for AC and registration require dedicated processors and are not straightforward for practical implementation in a clinical setting. Moreover, all AC map registration methods have still produced regional PET images with attenuation of > 2.4%, and hence, the PET quantification issue remains unresolved when using the standard arrays.

Knowing the effect of the vendor’s standard array (flexible body-matrix) on PET quantification, there is an urgent need to develop and validate a PET-compatible cardiac array that does not require hardware attenuation correction.

In a previous study, we assessed a novel 32-channel phased array, prospectively designed for PET/MRI cardiovascular imaging, and reported that it was capable of achieving high spatial and temporal resolution (up to acceleration factor *R* = 6) for MRI (Farag et al., 2019).

In the present study, the same novel array is thoroughly evaluated for PET image quality and compared to two standard arrays. Hardware AC maps for the posterior part of each array were developed together with the NEMA-phantom AC map. In order to quantify the effect of each part of the array on PET counts separately, NEMA Standard Publication NU 2-2001 for image quality measurements (NEMA, 2001) were performed using the anterior, posterior and both anterior/posterior parts of each of the three arrays, as well as, for no-array. Applying attenuation correction during image reconstruction was performed using two approaches: (1) MRAC, where PET image reconstruction used MRI-based AC map for the liquid in phantom, produced by the scanner, together with a dual-energy CT-based AC map for the housing of the NEMA Body-Phantom, and (2) DCTAC, where reconstruction of the PET image used dual-energy CT-based AC maps for both the liquid in the phantom and the housing of the phantom. Although we report using two reconstruction approaches, the scope of the study is not to compare the AC approaches. Instead, the two approaches were used to demonstrate the PET performance of the novel array in the best-case scenario of attenuation correction (DCTAC) and conventional PET reconstruction (MRAC) at low resolution.

## Materials and methods

The method and some of the materials described here are adapted from NEMA Standard Publication NU 2-2001 for PET image quality and accuracy of attenuation (NEMA, 2001), which has been used for assessing image quality of PET systems and instrumentation (Daube-Witherspoon et al., 2002; Ziegler et al., 2015).

### The arrays under evaluation and phantom

**Fig. 1:**
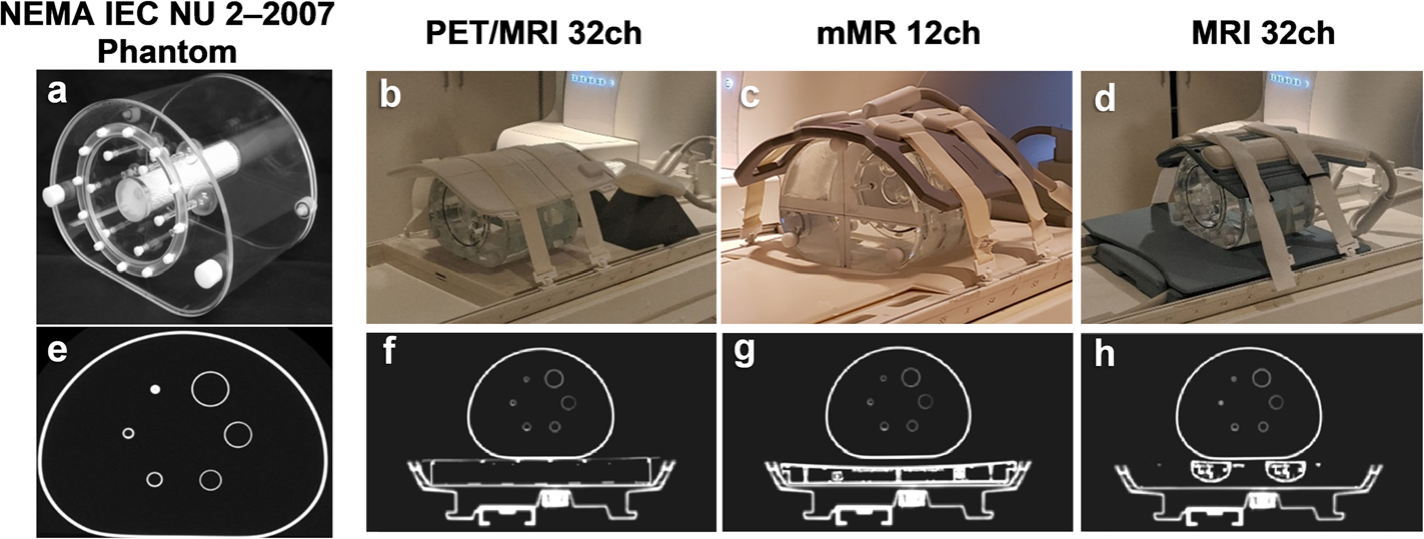
NEMA body phantom (**a**) together with the novel PET/MRI 32ch, standard mMR12ch and MRI 32ch arrays (**b**, **c** and **d** respectively). The DCTAC maps for each hardware showing here the axial central slice for the NEMA phantom with posterior view of PET/MRI 32ch, mMR12ch and MRI 32ch arrays (**e, f**, **g** and h respectively). All u-maps are windowed to the same level

A NEMA Body phantom IEC-2007 (National Electrical, Manufacturers Association Washington, DC, USA) was employed to carry out all PET quantification in this work (Fig. 1a). As shown in Fig. 1 b, c and d, the three arrays used in this study are as follows: the novel 32-channel PET/MRI array (Ceresensa, Canada 2016) consisting of two parts, anterior (flexible) and posterior (fixed); the 12-channel mMR array (Siemens Healthcare Limited, Erlangen, Germany) comprised of a 6-channel body matrix (flexible anterior) and 6-channel spine-matrix (fixed posterior); and the standard 32-channel MRI array (In-Vivo Corporation, Gainesville, FL, USA) with anterior (flexible) and posterior (fixed) parts.

### AC maps

In order to achieve accurate PET quantification, AC maps for all hardware within the scanner must be developed and applied during attenuation correction; this includes the NEMA phantom housing (i.e. phantom without its liquid content). Therefore, we developed all necessary hardware AC maps using a Dual Energy CT (GE Healthcare, Discovery CT750 HD, Waukesha, USA) scan of the hardware components following the method of bilinear transformation of Hounsfield units (using mono-energetic reconstruction at 70 keV and 140 keV) to 511 keV linear attenuation coefficient (Carney et al., 2006). The developed DCTAC maps were for both the empty and filled NEMA body phantom and the posterior part of each array, while the spine-matrix array was excluded, since its AC map is provided by the vendor.

For non-hardware AC maps, such as that related to the de-ionized water included in the NEMA phantom, two scenarios were examined: (1) rely on MRAC to correct for water attenuation, with the rationale that this is the scenario of a non-research/ clinical study where pre-acquired patient CT data is not normally available to the PET/MRI system, and (2) develop DCTAC map for the phantom filled with water and use it for off-line attenuation correction of the PET image, with the rationale that this is the scenario of a research setting, where patient PET/CT or CT-only data could be available for offline reconstruction.

### PET phantom preparation and measurements’ set-up

The phantom background is defined to be the phantom volume without the six spherical inserts nor the lung insert. The phantom background (volume ~10.2 L) was filled with deionized water and injected with ^18^ F-FDG to reach an activity concentration of 5.3 ± 0.1 kBq/ml. All six spheres (with inner diameters 10, 13, 17, 22, 28 and 37 mm) were filled with ^18^F-FDG to reach an activity concentration of 201 ± 5 kBq/ml, making the phantom activities reach a 1:38 background-to-sphere ratio, where the background activity corresponds to a typical injected dose for total body studies (NEMA, 2001).

Markers were produced on the patient table, as well as the phantom body, and when both markers were matched, the same position of the phantom on the table was consistently achieved with ± 1 mm tolerance. During all measurements, the isocentre was selected to be at the centre line between position number 4 and 5 of the spine-matrix (marked on the patient table). This set-up ensured that no additional objects employed for positioning during the PET measurement, and hence, data are free from added errors. The set-up also ensured the elimination of attenuation variation that the patient table itself may contribute.

### Image acquisition and attenuation correction

For ease of reading throughout this manuscript, the anterior part of an array is denoted with letter A while the posterior part is denoted with letter P, and therefore, both parts of an array are denoted with A&P, and part(s) may be referred to as case(s)**.**

All PET acquisitions were performed on a 3.0-T PET/MRI system (Biograph mMR Software Version VE11P, Siemens Healthineers, Erlangen, Germany) and followed the identical workflow routine. Evaluation of one complete array required one imaging session consisting of four PET measurements, where the first measurement was started once the activity reached the concentration described above. Each measurement comprised of a Dixon MRAC acquisition (2.5 min) and a PET acquisition (10 min). The four PET measurements were carried out in the following order: (1) the complete array (A&P) and NEMA phantom, (2) the posterior part (P) of the same array and NEMA phantom, (3) the anterior part (A) of the same array and NEMA phantom and (4) the NEMA phantom with no-array. Two imaging sessions were performed for each array, yielding two data sets for data analysis.

#### MRAC

By MRAC, here, we refer to the PET/MR scanning conditions where only the posterior part of the array and the patient table hardware AC maps, together with MR-based AC map of the liquid in the phantom, are used for PET attenuation correction during image reconstruction. A MRAC map of the liquid portion of the NEMA phantom was acquired with the manufacturer’s standard 2-point Dixon (3D dual-echo spoiled gradient sequence) using TR/TE = 4.14/2.51 ms, Matrix size = 240 × 126 × 127 at a resolution of 2.08 × 2.08 × 2.02 mm^3^ and is intrinsically registered to the PET images. The AC maps of each array’s posterior part along with the NEMA phantom housing were added to the scanner. For clarity, the reconstruction of the no-array AC images on the scanner used the phantom housing and patient table hardware AC maps, together with the phantom water MRAC map. The Biograph mMR’s standard PET reconstruction algorithm, an Ordinary Poisson Ordered Subsets Expectation Maximization (OP-OSEM) (Comtat et al., 2004) approach, was applied using 3 iterations, 21 subsets and a 4-mm Gaussian filter.

#### DCTAC

In order to obtain the ideal AC for both the hardware and liquid portion of the NEMA phantom, the raw, nonattenuation-corrected (NAC) data of each measurement was reconstructed offline using DCTAC of the NEMA phantom including the liquid portion and other hardware DCTAC maps. All PET reconstructions were performed offline using the manufacturer’s e7-tools (Siemens Molecular Imaging, Knoxville, USA). The PET images were reconstructed using the same algorithm and parameters as described under the MRAC section above. The PET images’ output matrix size was 344 × 344 × 127 to achieve ideal reconstruction modality (Ziegler et al., 2015).

### Data analysis

All data were corrected for the PET decay, due to the time used for hardware changing and sequence set-up, scatter and attenuation, before carrying out the analysis. All image processing and data analysis were computed using Matlab 9.3.0 (The MathWorks, Natick, MA, USA).

#### NAC data

NAC data are important in this analysis since it does not include any errors that an AC process might introduce. From NAC data, the global mean and standard deviation of each masked image (slice) were estimated. This mean and standard deviation were estimated for all cases of the three arrays, as well as for the no-array. The no-array data was considered the frame of reference that each part of an array was compared to. Therefore, the RPD between no-array and each part of an array was estimated and reported using equation 4 as reported in (Farag et al., 2019) and presented here for convenience, where *v*_1_ is the no-array case and *v*_2_ is any given case (A, P, or A&P).

$$ \mathrm{RPD}=\frac{v_1-{v}_2}{\ 0.5\ \left({v}_1+{v}_2\right)}\ 100\% $$

#### AC data

According to the NEMA NU 2–2001 standard, PET image quality can be analysed from parameters such as CR, BV and CNR. Therefore, each sphere’s CR, BV and CNR were estimated from the relationships described in (NEMA, 2001), and the mean and standard deviation for each array and for no-array were computed and reported. Relative percentage difference maps between PET images from no-array and images from each array part were computed. PET image quality parameters were estimated for both scenarios of AC data, images reconstructed by the scanner and images reconstructed offline using e7-tools. Statistical evaluation of the resulting means used a one-factor analysis of variance (ANOVA) with ‘array’ as the between-subject factor (alpha = 0.05). In the null hypothesis, no significant difference exists between all three arrays (equal means), and in the alternative hypothesis, means are significantly different with no a priori direction. The test was applied on the data from each array part (A, P and A&P), and the degree of freedom, *F*-factor, and *p* values are reported.

## Results

This study focused on assessing each part of the novel array independently for global and regional attenuation through PET image quality indicators and compared it to the no-array data as a frame of reference. The study particularly aimed to quantify the attenuation effects of the flexible (anterior) part of the array, since registration of its AC map is typically considered a great challenge likely to be alleviated by the use of essentially PET-transparent anterior parts.

### AC maps

The NEMA phantom is shown in Fig. 1a, and the array under evaluation is shown in Fig. 1b together with the two standard arrays (Fig. 1c, d). Figure 1 e, f and h demonstrate the in-house developed DCTAC maps for the NEMA phantom, the novel 32ch posterior and the MRI 32ch posterior array respectively, while Fig. 1g shows the spine-matrix AC map provided by the vendor. All posterior AC maps are shown with added patient table and NEMA phantom map which are used during PET image reconstruction on the scanner.

### Global means

#### NEMA phantom NAC data

**Fig. 2:**
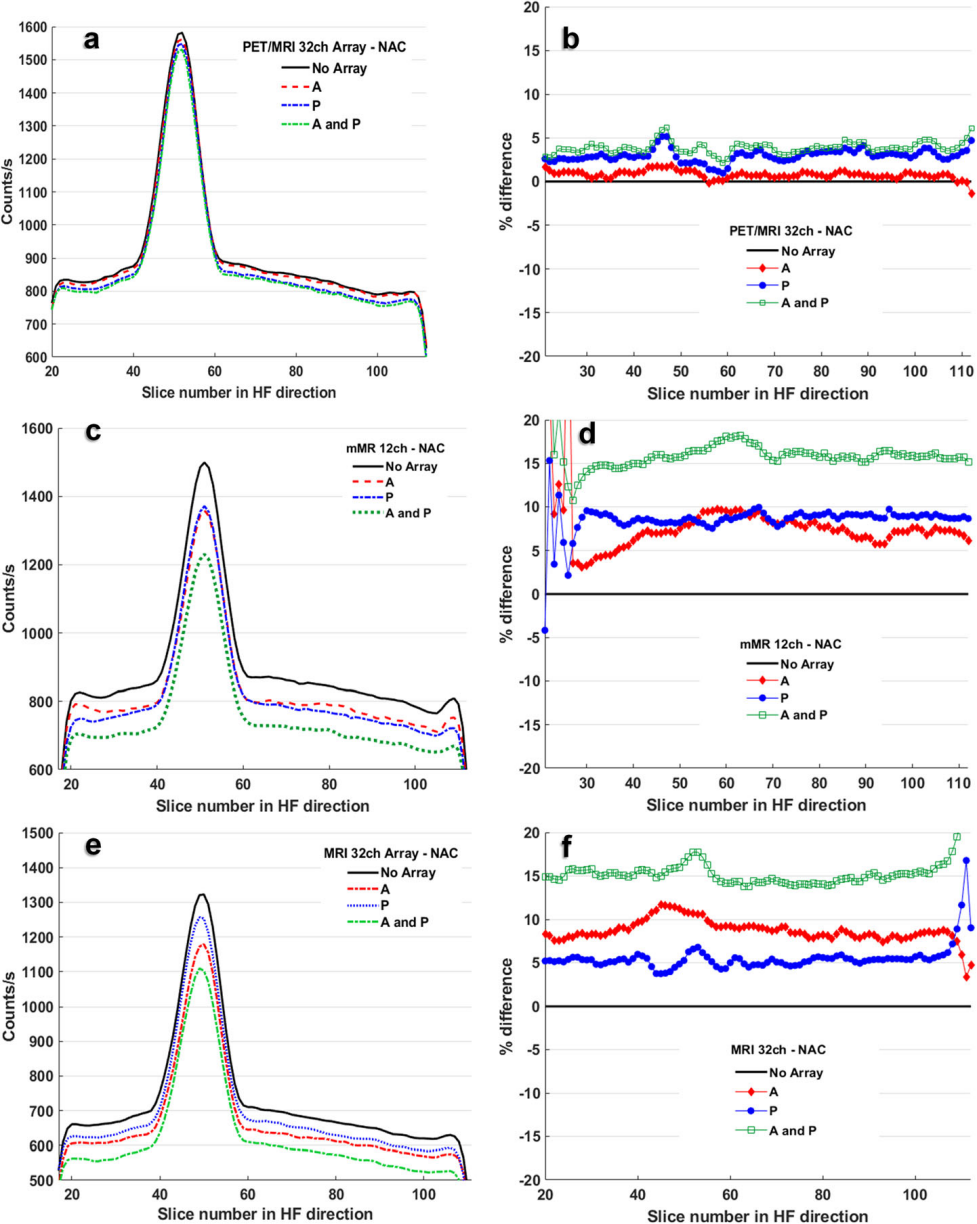
NAC data are shown in the left column of the figure, with plots showing as counts per second vs the acquisition plane number for all cases A, P, A&P and no-array. Plots are for (**a**) the novel PET/MRI 32ch, (**c**) the mMR 12ch and (**e**) the MRI 32ch arrays. The right column includes the corresponding RPD plots for each array (**b**, **d** and **f**) at case A, P and A&P, which resulted from estimation of difference between a mean of an image for each case and mean of its matching image from no-array

Figure 2 a, c and e show the NAC data acquired for each of the three arrays PET/MRI 32ch (novel), mMR 12ch and MRI 32ch respectively, where the decay-corrected mean of each image counts per second (CPS) in the *z*-plane are plotted, for each array parts A, P, A&P and no-array. The RPD between each part of an array and no-array are shown in Fig. 2 b, d and f. It is observed that the novel array demonstrated a steady RPD < 5% in all cases of A, P and A&P. The RPD for both standard arrays varied between 4 and 19%. The detailed RPD of the global mean for each array and its parts are reported in Table 1.

**Table 1:** RPD of the global means for each case A, P and A&P; NAC, reconstructed with MRAC and DCTAC. The negative value represents over-correction of attenuation

		A	P	A&P
PET/MRI 32ch	*NAC%*	1.9 ± 0.6	3.0 ± 0.7	4.3 ± 0.7
	*MRAC%*	2.1 ± 0.8	0.7 ± 3.1	1.5 ± 3.9
	*DCTAC%*	1.9 ± 0.9	− 1.8 ± 3.5	0.0 ± 2.5
mMR 12ch	*NAC%*	7.3 ± 2.3	8.7 ± 1.3	15.6 ± 1.4
	*MRAC%*	4.8 ± 0.9	10.7 ± 3.6	13.4 ± 3.4
	*DCTAC%*	4.6 ± 1.2	− 3.7 ± 2.8	2.8 ± 2.6
MRI 32ch	*NAC%*	8.9 ± 1.5	5.8 ± 2.2	15.3 ± 1.9
	*MRAC%*	8.3 ± 0.9	5.6 ± 1.5	7.4 ± 8.2
	*DCTAC%*	7.9 ± 1.3	− 2.4 ± 3.6	9.5 ± 2.7
*p value*	*MRAC*	0.001	0.014	0.008
	*DCTAC*	0.001	0.050	0.121

#### MRAC

In Table 1, RPD of global means are also reported for each case of each array, where RPD for A&P-MRAC case for the novel array is estimated to be 1.5% compared to 13.4% and 7.4% for the same case with the mMR 12ch and MRI 32ch respectively.

**Fig. 3:**
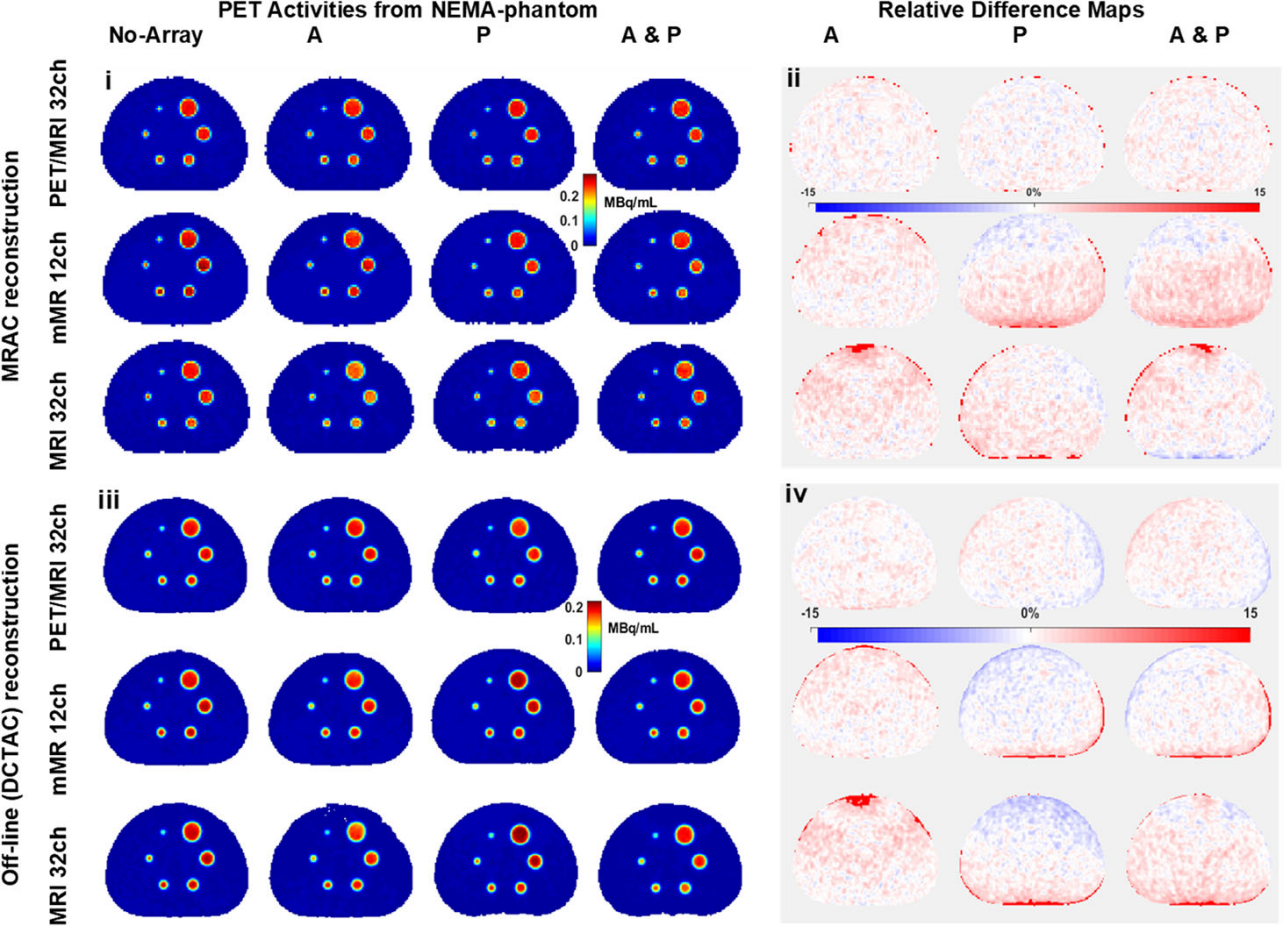
Reconstructed AC images from all cases (no-array, A, P, A&P) of the NEMA-phantom using both; (*i*) MRAC matrix = 172 × 172 and (*iii*) DCTAC matrix = 344 × 344. The images in (*ii*) and (*iv*) are the RPD maps corresponding to MRAC and DCTAC reconstructions respectively, which are result from the estimated difference between each case (A, P and A&P) with the no-array in a pixel-by-pixel fashion

Figure 3 (i) shows in the MRAC-constructed PET images obtained on the NEMA phantom with the four cases no-array, A, P and A&P labelled for each array, all spheres are visible. In Fig. 3 (ii), RPD maps are shown for the three arrays, which are the results of comparing each case of an array with no-array, by means of percentage difference by pixel-by-pixel approach. In Fig. 3 (ii) also, the RPD from the anterior part of the novel array ranged from − 0.5 to 1.0%, while the RPD from mMR 12ch and MRI 32ch ranged from − 1.4 to 19.3%.

#### DCTAC

The global mean of RPD between each case vs no-array using the DCTAC reconstruction are reported in Table 1. The RPD of global means, for both mMR 12ch and MRI 32ch, in case P, are − 3.7% and − 2.4% respectively, while for the novel array it is − 1.8%. The A&P case for the novel array produced a global mean RPD of 0.0%, compared to 2.8% and 9.5% for the standard arrays. In Fig. 3 (iii), the NEMA phantom images reconstructed using the DCTAC for all cases as well as no-array with all spheres showing. Fig. 3 (iv) shows the RPD maps for all cases of each array resulted from their DCTAC data, where the lowest regional variations belong to the novel array A case. The RPD from both mMR 12ch and MRI 32ch in Fig. 3 iv shows high regional variation in case A (close to the top of the RPD maps), which was not the case of the novel array.

### Image quality

**Fig. 4:**
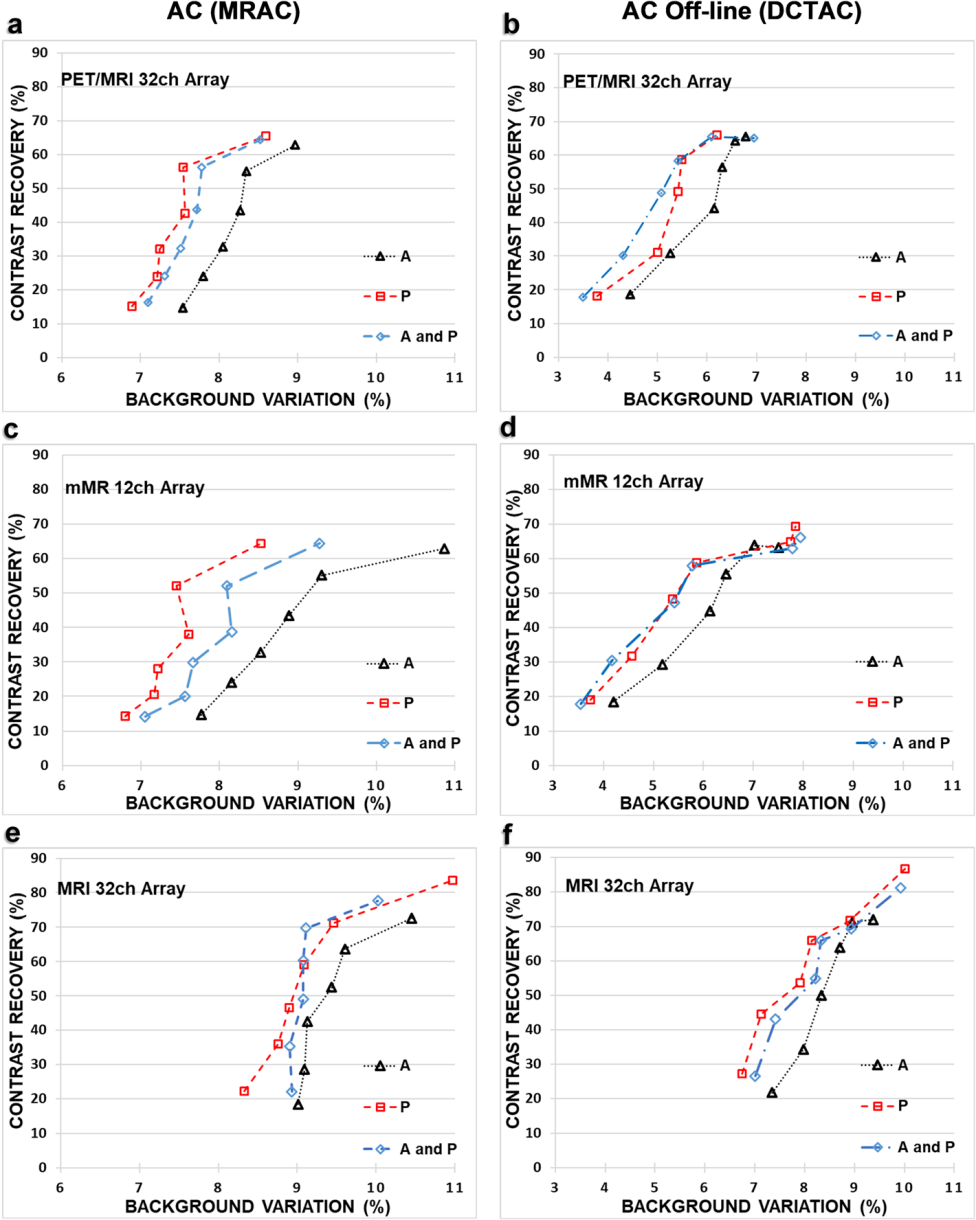
CR vs. BV (from six spheres) for each array as PET reconstructed using both MRAC (**a**, **c** and **e**) and DCTAC (**b**, **d** and **f**). First data point represents the 10-mm sphere, while the last data point represents the 37-mm sphere. The A, P and A&P cases are plotted in dotted, dashed and dash-dotted lines respectively

PET image quality parameters CR and BV using the MRAC reconstructions, for each array and each case, are shown in Fig. 4 a, c and e. The BV with the smallest range and magnitude was found in the novel array. The CR for case P of the MRI 32ch array is measured to be 83.6% for the 37-mm sphere, superseding the contrast recovered by no-array for the same sphere size. From the MRAC data, the percentage of BV for the MRI 32ch were found to be in a range between 8.3 and 9.0% for the 10-mm sphere and between 10.0 and 11.0% for the 37-mm sphere, which were higher compared to the novel array.

In Fig. 4 b, d and f, the image quality parameters BV estimated from DCTAC data are plotted for all parts of the arrays. BV from the A&P case of the novel array reported to be the lowest in all data.

#### Anterior analysis

Table 2 lists the relative differences between no-array CR and each array’s anterior (A case) for all spheres, where the lowest RPD for sphere sizes 10 mm, 22 mm and 37 mm using the MRAC data was for the novel array. The case A of the novel array recorded the lowest RPD across all sphere sizes, as seen from the DCTAC data in Table 2.

**Table 2:** CR relative percentage difference between no-array and the anterior (A) part of each array for each sphere size. The CR was estimated for both reconstruction methods (MRAC and off-line DCTAC)

	Sphere size (mm)	10	13	17	22	28	37
MRAC	PET/MRI 32ch (%)	4.8	2.8	5.2	3.8	2.1	− 1.5
	mMR12ch (%)	15.1	2.6	− 4.0	− 5.4	2.0	5.2
	MRI 32ch (%)	− 20.2	− 17.9	− 22.7	− 16.4	− 13.1	− 17.3
	*p* value	0.003	0.01	0.00	0.001	0.00	0.00
DCTAC	PET/MRI 32ch (%)	3.8	0.9	3.6	2.0	3.7	2.4
	mMR12ch (%)	6.0	8.0	4.1	3.5	4.2	6.6
	MRI 32ch (%)	− 10.6	− 7.7	− 9.5	− 11.1	− 6.8	− 6.2
	*p* value	0.007	0.011	0.129	0.053	0.050	0.001

**Fig. 5:**
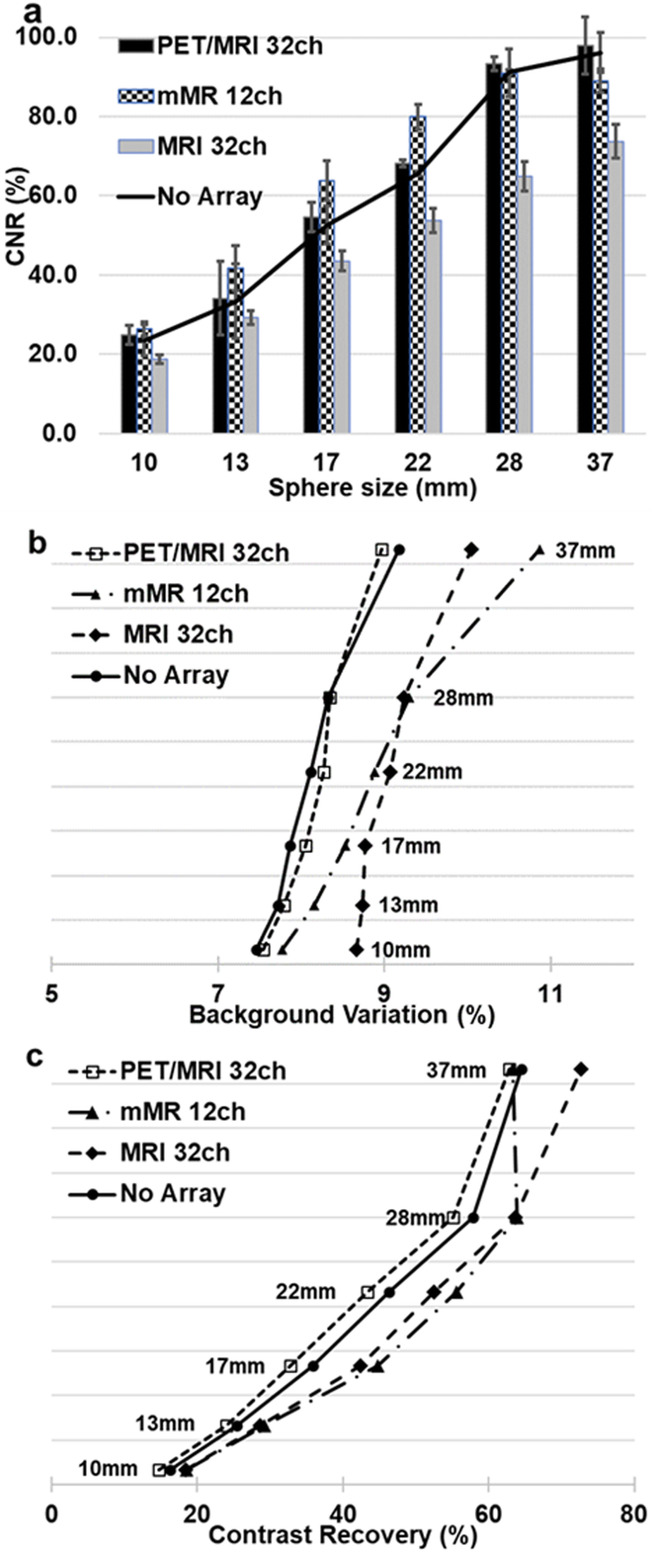
DCTAC data for each anterior component of the three arrays are compared to no-array for all spheres, (**a**) CNR, (**b**) BV and (**c**) percentage CR. The CNR measured when using the PET/MRI 32ch anterior was within 2% of the CNR values from no-array for all sphere sizes, while CNR from the MRI 32ch were the lowest for all spheres and values for mMR 12ch similar to the PET/MRI 32ch. Similar patterns were observed for both the CR and BV

Figure 5 a is a histogram combined with line plot (solid line plot) presenting the CNR from DCTAC data for case A of each array together with the no-array, as reference. The novel array anterior part is found to have the closest CNR values to the reference array for all sphere sizes. Both the MRI 32ch and the mMR 12ch surpasses, or are lower than, the no-array reference. The BV and CR from the MRAC data from case A for all sphere sizes are plotted in Fig. 5 b and c respectively. The BV produced by the novel array has recorded the closest values to the no-array data with BV ranging from 7.5% at 10-mm sphere to 8.9% at 37-mm sphere. Following the same pattern, the CR values were found to be the closest to no-array with range from 14.7 to 62.8%.

### Data analysis

Differences between mean activities for each case (A, P or A&P) of the three arrays were tested with 1 × 3 one-factor ANOVA using the array as a factor as reported in Tables 1 and 2. For the case A&P, there was no statistically significant difference between group means as determined by one-way ANOVA (*F*(2,6) = 3.0, *p* = 0.121). For the cases of A and P, there were statistically significant differences between group means as determined by ANOVA (*F*(2,6) = 33.1, *p* = 0.001) and (*F*(2,6) = 4.2, *p* = 0.050) respectively.

## Discussion

In this work, a novel 32-channel array prospectively designed for hybrid PET/MRI cardiovascular imaging was evaluated for 511 keV photon attenuation. The evaluation incorporated a comparison of the novel array PET performance measuring PET image quality parameters using the NEMA NU 2-2001 Body Phantom compared to a reference measurement (no-array). The novel array’s separate parts were also assessed individually and together and compared to its counterparts from two standard arrays currently used in PET/MRI cardiovascular imaging. Two different PET image reconstruction techniques (MRAC and DCTAC) were used to assess the novel array performance in the context of non-research (clinical) and research settings.

To date, research on hardware performance in PET/MRI environment, specifically RF cardiac phased arrays, has been focusing on flexible anterior part, since it is the most challenging for AC (Eldib et al., 2014; Kartmann et al., 2013; Paulus et al., 2012). Nevertheless, in this study, all configurations/settings of the array (A, P or two parts A&P) were evaluated for each of the three arrays. This was fair since the setting for simultaneous PET/MRI cardiovascular imaging utilizes both anterior and posterior parts of an array. However, we chose not to produce DCTAC maps for the anterior part of each array, nor to apply the AC maps during PET image AC reconstruction, in order to mimic the non-research setting (clinical).

Notably, the results obtained in this study for the anterior part of both standard arrays (mMR 12ch and MRI 32ch) are in agreement with previously reported results by Eldib et al. (2014), Kartmann et al. (2013) and Paulus et al. (2012). The anterior part only of the mMR 12ch array caused overall loss of counts of 4.7% (Table 1) and may cause up to 2.55% residual error after applying AC maps and utilizing specially developed algorithms to reconstruct PET images off-line, as reported by Paulus et al. (2012). Similarly, for the MRI 32ch phased array anterior case, it was reported by Eldib et al. (2014) that loss of true counts could be as high as 10%, while regionally estimated to be as high as 22% and as low as 2.7% globally, after correction using CT-based AC map and using a complex algorithm off-line. Both standard arrays anterior parts affected the PET images, which is emphasized by the high values in the RPD map and are visible in Fig. 3 (ii and iv). These effects are found to be in agreement with the results reported by Kartmann et al. (2013).

In this study, we have shown that the novel array anterior has a superior performance (≤ 2% RPD) with no attenuation correction maps applied, while both A&P attenuates less than 2.5% together after posterior-correction. Although no AC-map for the anterior of the novel PET/MRI 32-channel array was used, the array had not affected the PET images, which was confirmed by RPD map homogeneity and low regional variation.

This, in comparison to its counterparts from the two standard arrays (mMR 12ch and MRI 32ch), is considered to be advantageous, in the sense that no registration is needed. Such advantage was significant (*p* = 0.001) for the anterior, though the A&P case for the three arrays’ difference was not significant (*p* = 0.121). At first, this may suggest that the attenuation from the complete A&P set for all arrays are not significantly different. However, upon a closer inspection AC data (Fig. 4d, e and f) of the P case and RPD maps (Fig. 3 (ii and iv)) for both standard arrays, one notices that the AC map of the posterior parts are over correcting, which is contradicted by the underestimated attenuation caused by the anterior part. Hence, AC map of the anterior part is necessary when using the A&P set for better global attenuation estimation. Considering the background variability as a representation of PET image homogeneity, the novel array can achieve the highest image homogeneity, since it produced the lowest BV. The BV is also a measure of statistical noise in the image confirming that the novel array causes the least noise (e.g. from inaccurate attenuation correction or poor convergence during iterative reconstruction).

In Fig. 3 (ii and iv), the high percentage, up to 15%, of attenuation observed (in the top of the phantom in the A cases, and the bottom of the phantom in the P cases) are due to a solid angle effect, where more annihilation photons are being attenuated or scattered near the array. When comparing PET images reconstructed with DCTAC vs. MRAC, case A shows better image homogeneity by approximately 1% for all three arrays. This could be explained by the increased accuracy of the CT-based map of the water in the phantom over the MR-based map of the same, which is susceptible to RF inhomogeneities, especially with the NEMA standard body phantom at 3.0 T MRI (Ziegler et al., 2013).

In the P cases of the mMR 12ch and MRI 32ch arrays, RPD maps suggest overestimation by 3 to 6% at the phantom top in areas nearest to the anterior portion of the array, while this is not present in the A&P cases for the same arrays. The overestimation could be due to inaccurate attenuation coefficients from the posterior AC-map, due to beam hardening artifacts from metal parts of the array and presented in the CT data. This could also be due to bias of the conversion model of HU to LAC at 511 keV in DCTAC. The absence of this overcorrection in the A&P cases could be explained by the presence of the anterior part during this scan and its under-correction effect, which is compensated by the overcorrection related to the posterior portion regions at the phantom top. Such a pattern is consistent for both mMR 12ch and MRI 32ch arrays.

## Conclusions

This study presented the PET performance of a novel 32ch RF phased array, prospectively designed for hybrid PET/MRI cardiovascular imaging. Although the novel array has 32 elements (almost 3 times more than the mMR 12ch), yet, we have shown that the novel array had better attenuation qualities that resulted in better PET images. The results from this study together with MRI performance of the same array reported in Farag et al. (2019) suggest its suitability for PET/MRI cardiovascular imaging. This novel array can operate without needing AC for the anterior part, simplifying the attenuation correction while at the same time improving PET image quality.

## Data Availability

The data that support the findings of this study are available from Lawson Health Research, but restrictions apply to the availability of these data, which were used under license for the current study and so are not publicly available. Data are however available from the authors upon reasonable request and with permission of Lawson Health Research.

## Abbreviations

PET: Positron emission tomography
MRI: Magnetic resonance imaging
FDG: ^18^F-Fluoro-deoxy-glucose
FOV: Field-of-view
RF: Radio frequency
SUV: Standardized uptake value
NAC: Nonattenuation-corrected
AC: Attenuation correction
LAC: Linear attenuation coefficients
UTE: Ultra-short echo time
CR: Contrast recovery
BV: Background variability
CNR: Contrast-to-noise ratio
SNR: Signal-to-noise ratio
MRAC: Magnetic resonance attenuation correction
DCT: Dual-energy computed tomography
SD: Standard deviation
OP-OSEM: 3i 24s Ordinary Poison estimation method
CPS: Counts per second
